# Text Books

**Published:** 1884-10

**Authors:** 


### TEXT BOOKS.

There are certain reference books that are essential to students entering college. They are needed for every-day use, to enable one to become thoroughly familiar with all the technicalities of a subject. Of course it is presupposed that works like that of Taft on Operative Dentistry, Garretson on Oral Surgery, Richardson on Mechanical Dentistry, Kingsley on Oral Deformities, with other standard text books, will have been thoroughly mastered, but there is a necessity for compendiums; condensed epitomes for pocket use and for every-day reference. One of the best of these, and that too upon a subject with which dental students are not usually sufficiently familiar, is Prof. Stubblefield's Handbook of Materia Medica and Therapeutics. The system upon which it is founded is an admirable one, and we can heartily commend it to every student.

